# Research is a Passion and not a Penance

**DOI:** 10.1055/s-0045-1810108

**Published:** 2025-08-29

**Authors:** Visweswar Bhattacharya

**Affiliations:** 1 Department of Plastic Surgery, Institute of Medical Sciences, Banaras Hindu University, Varanasi, Uttar Pradesh, India

This article is about my learning and experience through more than four decades of intense clinical research. My initial lessons in animal experimentation, imparted by my mentor Prof. K. N. Udupa, founder Director of Institute of Medical Sciences, Banaras Hindu University, were learned from 1973 onwards, during my general surgery postgraduation era, in surgical research laboratory. Prof. Udupa ignited the spark within me by instilling the importance of research, which I pursued all through my carrier. I progressively realized that maturity is not when we start speaking big things but when we start understanding small things. It is not necessary to have a latest updated laboratory to perform clinical research but it is a mind to generate thoughts capable of assuming incredible proportions with the help of technology. The purpose of this writing is to replace reluctance by stimulation to make our members try their hand at research by igniting the research spark present in each one of us. Genesis of thoughts could be through failures or an accidental successful innovation. It may be a result of disappointment by analyzing one's own result or patient's dissatisfaction and criticism. The weight of the word “research” is not to be feared but undertaken to gain an advantage.

Research as the term implies means to search again with a novel thought or idea in mind to discover something new and innovative. The discovery, however, should advance convincingly an existing body of knowledge in a specified field or modify the understanding of an already existing theory of a procedure via various interrelated parameters designed and based on the original thoughts of a scientist. It is important to realize that advances in knowledge and technology are deep rooted in basic research. It is a consequence of one's interest to upgrade the existing knowledge in a particular area. It helps to modify or to establish the understanding of fundamental basics related to common clinical implications. It also helps one to learn the usage of appropriate scientific words with better expression during writing and presentation. As we have abundant material, we should utilize, propagate, and receive wide acclaim.

Research is a tedious job but at the same time enjoyable too. Man by nature is curious and though the research instinct is present in each one of us, it is not a general phenomenon. Inspiration and some special circumstances trigger and compel analytical minds to leap into the unknown with the hope of finding something new. Illustrious researchers with commendable findings often are a strong source of inspiration serving as a model for amateurs venturing in the field. Since it's a daunting task, research should be undertaken by only those who have a strong inclination for it because it requires patience, skill, devotion, and dedication. However, any person can venture without any phobia. Successful research is passion-oriented. It should be a pleasure not a penance because when the undertaking yields a successful parameter the passion turns into an obsession for further study and findings. To start with a moderately equipped laboratory, to perform clinical research is a basic requirement. However, a latest updated one is always advantageous.

Consistency is a very important attribute which dedicated researchers should possess. Adopting a logical pathway, hypothesizing a thought is the first thing which should germinate in a scientist's mind. Once the parameters to work on are decided, the choice of a proper team drawn from various departments, relating to the specific field is a mandatory requisite, followed by a thorough discussion with teammates on the topic to formulate a study plan, inclusive of experimental aspects for standardizing the techniques and for the application of results in human beings, respectively. Clearance from the ethical committee is extremely essential prior to starting the work.

The findings of a parameter require analysis as a result of which further parameters gradually unfold. After successful completion of studies on a particular aspect an inference is drawn proving the rationality of a procedure. A hypothesis thus becomes a theory. The technique becomes applicable and capable of being popularized in clinical conditions. Approved and accepted by the scientific world it becomes the discovery of a scientist.

*Genesis of thought* : A thought can be evoked by many different situations.

(a) It could be through failures.
(b) An accidental successful innovation.
(c) One may encounter plenty of specific clinical material without any definite acceptable solution. He/she may have adopted certain techniques to deal with them for a long time without satisfactory results. Their dissatisfaction may have persuaded them to search further for a better rational technique.
(d) Too many unconvincing procedures for a given problem, not based on sound principle, and therefore not well explained may require further explanation.
(e) Attentive listening in a conference where the subject is open to controversy gives birth to new ideas demanding reasoning.
(f) Scanty literature on a common clinical entity can trigger the thought process.
(g) A part of an ongoing but unfinished research can also prove to be a source for further research.

*Selection of topic* : The topic of research is of utmost importance. It should be an area of one's interest, preferably, a combination of basic research and clinical application. A project of this sort draws the attention of a wide spectrum of people. However, before finalizing a topic one should be well aware of its feasibility in respect to (a) laboratory facility, (b) man power, (c) experimental work place if needed, and (d) clinical material. All these are important as they have a direct bearing on the topic because the parameters will have to be planned accordingly.

*Feasibility* : At times the work has to be done outside duty hours or even on holidays as the concerned coresearchers or the equipment may not be available during routine hours. Therefore, before thinking about any parameter, one must have first-hand knowledge regarding the full-hearted cooperation and involvement of the coresearchers, the other members of the team, and the availability of equipment. The outcome of the first parameter may necessitate a few more observations in the same department. A research, therefore, must ensure consistent involvement with teammates by regularly interacting with them, while never quite forgetting to give them full credit for their endeavor and enthusiasm.

*Theme of research* : It can be a short project or a long one. Both can be worked on simultaneously. The area should have enough clinical material, laboratory and investigative facilities, and an analyzing team lead by the mentor. In the beginning three or four topics should be selected. After one or two years feasible selective topics can be taken up.

*Short* : A short project involves a study or modification of a particular surgical method where a single procedure needs standardization through the most effective and convincing solitary parameter.

*Long* : A long project is a farsighted project of a wide area of research extending over years requiring multiple parameters. Initially, few parameters are postulated and later their analysis eventually unfolds other relevant methods. Most of the time interdepartmental collaboration is necessary depending upon the nature of the procedure.

A precise concept of the area of research is necessary as the theme depends upon that. For example, if one intends to work on any reconstructive procedure then the theme should encompass the following issues:

(1) Structural analysis
(2) Functional analysis
(3) Clinical applications

The theme starts taking shape on the basis of these analytical findings compiled together.

*Sequence of thought* : Once the topic has been decided, it is important to give a serious thought with the mind at peace, regarding the sequence of procedures. Basically, it's the mind which generates thoughts. As imagination is more important than knowledge, it can assume incredible proportions with the help of technology.

Sequence means arranging several parameters in mind so that they follow in order, one after the other. Preferably, the basic scientific aspect should be taken up initially, as it is essential to realize that it is more difficult to deal with basic science than with the clinical aspect.

*Decision of initial parameters* : Technical advancements are adjuvant twigs only, human brain is the main trunk. Remember, most of the time, “simple is effective.” The selection of initial parameters should be such that the basic features of the hypothesis can be based on them. It should deal first with the structural aspect because on this foundation the other features of the theme will revolve. For example, when we decide to study about a reconstructive procedure, it should deal with the constituents of tissue and their blood supply. This can be best studied through meticulous cadaveric dissection. Such a study shall reveal what is not known. Thus, the findings will generate enormous interest in the subject. Based on the observations, further parameters can be designed. This will arouse the desire to study the constituents and the blood supply separately and in more detail, through different parameters. The enclosed petals of the subject will then gradually unfold revealing the hidden secrets. Thoughtful, early parameter is the key to success. If one works in an unplanned way, with a higher parameter, he/she will soon realize that there is no other option but to revert back. The progress and tempo of research will automatically be hampered.

*Formation of team* : Research is not a one man show. It involves team work which usually consists of two types of people: (1) who are oriented with the subject and (2) those who need to be oriented.

The first group consists of those who are of the same specialty, with active participation in the basic discussion relating to formulation of parameters. They are either colleagues or residents or laboratory technicians. They have self-interest and therefore are easy to induct.

The second group consists of variable individuals depending upon the parameter. They may be from varied branches of science, namely, paraclinical, basic science, or from a totally different faculty. It needs a lot of effort and persuasion for the main investigator to create interest, explaining the finer details and facing many irrelevant but inquisitive queries. Persistent effort ultimately succeeds to arouse involvement in them. A union of two groups leads to a joint effective venture. Flexibility is the key to success. Listen to others' views by encouraging discussion at every opportunity. Anybody around may provide new ideas.

They need to be convinced and involved to such an extent that they are eager to work in free time even on holidays when the ambience is peaceful because research requires peace of mind for the brain to function at a higher level.

*Review of literature* : Access to review of literature precisely in relevance to the parameters, is a must for research. An in-depth study of the existing data exposes the unexplored areas making the lacunae evident. The detailed study provides ideas regarding the basic methodology to be adopted, the type of study model to be used, as well as the limitations of some of the techniques. The question in the scientist's mind, “Can I find out the solution or add something new to the work” thus receives a positive answer.

The clinical application and utilization of knowledge, in the form of the outcome of the study, also come to the surface when existing material has been thoroughly perused. Comprehending and comparing the findings of other workers with one's own is of immense help in making one's finding credible. Articles therefore should be read methodically and reviewed with concentration.

*Visualize the sequence of steps* : Each parameter requires thoughtful concentration to proceed meticulously in proper sequence. Even the minutest detail should be paid attention to. The materials required to conduct a particular parameter must be enlisted and procured before starting. For example, if one plans to perform dye study on fresh cadaver then he/she should arrange for (a) all the surgical instruments, (b) dye and the injection tool, (c) good light source, and (d) good resolution camera with a suitable background for documentation. Without these essentials, the anticipated result may not be achieved and documentation may turn out to be unsatisfactory, requiring unnecessary repetition of the procedure.

*Method of documentation* : A credible piece of research needs proper documentation which should be done from the beginning and should be of excellent quality. Extensive documentation is better than scanty as it can be later edited as per requirement. It is essential for both presentation and publication. Failures and hurdles are an incredible part of research and instead of being discarded, they too should be documented because negative results also bear significance as no finding is irrelevant. No matter what happens, you will learn something. Science is not only about getting “the answer.” Knowing that something didn't work, is actually knowing quite a lot. Experiments that don't turn out as planned are an important step in finding an answer. We must analyze, identify, and rectify them by taking the steps required in future. At the end of the study, the documents should be scrutinized thoroughly and a discussion with teammates should be arranged, to avoid missing out of detail on any aspect. Gradually, over the years the material grows in quantity and quality ready for acceptance and publication in the scientific domain. The endeavor then becomes an experience of originality for the scientist.

*Analysis of findings* : Results should not be prefabricated; instead, the mind should be open to analysis and acceptance of findings. After a preliminary analysis of the findings the whole team should critically evaluate the results. This certainly provides an unbiased opinion and opens up several windows for further investigation. It also demarcates the limitation of conclusion in a particular work. At the same time, it decides what other parameters should be studied to come to a full-proof inference for safe and successful clinical application. Can any new or modified technique be arrived at?

One successful research spurs you on to undertake many more, broadening your horizon in a particular field. There may be contradiction among the team members leading to repetition of the method. Contradictions should be welcomed as they lead to a better understanding of the subject.

*Clinical applications* : The standardization in animal experimental model is essential for research with clinical implication as its objective. This also helps in getting clearance from the ethical committee. Therefore, one should preferably have access to animal experimental research laboratory.

In two situations such research is needed:

1. When there are several unsatisfactory techniques for a given problem.
2. When individual surgeons deal in their own way changing the procedure constantly.

A technique based on sound rationality with the potential to be practiced by majority of hands should be evolved. This is only possible through research with clinical implication.

*Subject presentation* : The most important characteristic of good research lies in its presentation. The outcome of study therefore should be presented at proper scientific forums regularly. The task is demanding and requires tremendous effort and devotion to make it of quality in terms of content and message. To make this possible the matter should be original or an addition throwing light on an already existing knowledge. Direct involvement in a project, with first-hand knowledge of the subject makes for authenticity. Prior to presentation, speculation of the queries that may arise after presentation is a must for a good researcher to prove his/her point of view. Thus, the satisfactory index of research must be kept high for maintaining world-class standards.

*Interaction* : A good research presentation is bound to generate lot of questions. The more one interacts the more one gains in terms of knowledge, which one can apply to one's work. Those who participate in interaction either by approving or contradicting need to be acknowledged because both help in increasing knowledge, throwing up new ideas and suggestions.

*Publications* : Publication is an integral part of research. Writing an article is an art. It becomes effective and worthy of being appreciated when the matter is a strong blend of basic research and appropriate clinical application. There should be subdivisions dealing with major aspects of the adopted procedure and its application. The language should be simple and an attempt should be made to publish it in subject-oriented, reputed indexed journals. Presentation and publication is what makes a researcher successful and known in the realm of science. Therefore, it is the duty of the innovator to stimulate subordinates and colleagues who sometimes give excuses as they are reluctant to write or are hesitant because their writing and communication skills are not developed. As a result, their qualities start fading and they become source of discouragement for others. They gradually get autoamputated from academics and the scientific world.

## Conclusion

Knowledge is unlimited. It is like a fathomless ocean. The deeper you strike the more treasures you discover. Science has limitless mysteries. Researchers are the ones who solve them bringing about a revolutionary effect by their thirst and quest. They face and overcome hurdles and experience immense pleasure and satisfaction with knowledge, untapped before, as their reward. The obsession becomes a passion. The mind is a humming beehive assailed by diverse thoughts. One never knows when he or she may experience the research sting.

This poem has been composed by Dr. Shipra Bhattacharya encompassing the whole theme of research.

RESEARCH SPARK

The essence of research lies in imagination

It is an idea in germination

Spurring minds of a generation

To indulge in exploration.

Though, not an easy occupation

It requires a team through collaboration

To work in cohesion

For its propagation.

Undeterred by reprobation

Instead, with heightened determination

And persistent experimentation

It establishes

The innovation

With document

For publication

In the global denomination.

